# Machine learning‐assisted point‐of‐care diagnostics for cardiovascular healthcare

**DOI:** 10.1002/btm2.70002

**Published:** 2025-02-03

**Authors:** Kaidong Wang, Bing Tan, Xinfei Wang, Shicheng Qiu, Qiuping Zhang, Shaolei Wang, Ying‐Tzu Yen, Nan Jing, Changming Liu, Xuxu Chen, Shichang Liu, Yan Yu

**Affiliations:** ^1^ Division of Cardiology, Department of Medicine, David Geffen School of Medicine University of California Los Angeles Los Angeles California USA; ^2^ Department of Bioengineering, Henry Samueli School of Engineering and Applied Science University of California Los Angeles Los Angeles California USA; ^3^ Department of Spine Surgery, The Third Hospital of Mianyang Sichuan Mental Health Center Mianyang China; ^4^ Department of Electronic and Computer Engineering The Hong Kong University of Science and Technology Hong Kong China; ^5^ Postdoctoral Research Workstation Chongqing Orthopedic Hospital of Traditional Chinese Medicine Chongqing China; ^6^ Department of Pathology and Laboratory Medicine, David Geffen School of Medicine University of California Los Angeles Los Angeles California USA; ^7^ California NanoSystems Institute, Crump Institute for Molecular Imaging, Department of Molecular and Medical Pharmacology University of California Los Angeles Los Angeles California USA; ^8^ Department of Nutrition University of California Davis Davis California USA; ^9^ Department of Computer Engineering, School of Engineering and Applied Science University of Virginia Charlottesville Virginia USA; ^10^ Honghui Hospital Xi'an Jiaotong University Xi'an China

**Keywords:** cardiovascular diseases (CVDs), continuous health monitoring, deep learning, machine learning, point‐of‐care (POC) diagnostics

## Abstract

Cardiovascular diseases (CVDs) continue to drive global mortality rates, underscoring an urgent need for advancements in healthcare solutions. The development of point‐of‐care (POC) devices that provide rapid diagnostic services near patients has garnered substantial attention, especially as traditional healthcare systems face challenges such as delayed diagnoses, inadequate care, and rising medical costs. The advancement of machine learning techniques has sparked considerable interest in medical research and engineering, offering ways to enhance diagnostic accuracy and relevance. Improved data interoperability and seamless connectivity could enable real‐time, continuous monitoring of cardiovascular health. Recent breakthroughs in computing power and algorithmic design, particularly deep learning frameworks that emulate neural processes, have revolutionized POC devices for CVDs, enabling more frequent detection of abnormalities and automated, expert‐level diagnosis. However, challenges such as data privacy concerns and biases in dataset representation continue to hinder clinical integration. Despite these barriers, the translational potential of machine learning‐assisted POC devices presents significant opportunities for advancement in CVDs healthcare.

**Translational Impact Statement**

Machine learning‐assisted point‐of‐care (POC) devices hold transformative potential in addressing cardiovascular diseases (CVDs), the leading global cause of mortality. These devices enhance diagnostic accuracy, enable real‐time health monitoring, and address challenges such as delayed diagnoses and high costs in traditional healthcare. Advances in deep learning frameworks enable automated, expert‐level diagnostics, paving the way for more accessible and personalized care. Despite challenges like data privacy concerns, these advancements underscore the clinical and translational significance of POC devices in improving cardiovascular health outcomes.

## 1 INTRODUCTION

Cardiovascular diseases (CVDs) remain the leading cause of global mortality, accounting for approximately one‐third of deaths worldwide.^1^, ^2^ Estimates suggest this figure could rise to 23.6 million CVD‐related deaths by 2030.^1^, ^2^ A significant portion of these conditions, such as arteriosclerosis, myocardial infarction, coronary heart disease, and hypertension, can be effectively managed or prevented with timely diagnosis and continuous monitoring.^3^, ^4^, ^5^ In addition, although diabetes is not classified as a CVD but is a significant risk factor for developing CVDs.^6^, ^7^ While diabetes is a metabolic condition characterized by elevated blood sugar levels due to insufficient insulin or ineffective insulin use, it contributes to cardiovascular complications by damaging blood vessels and nerves that regulate the heart and circulation. This damage accelerates atherosclerosis, increases the risk of hypertension and dyslipidemia, and makes individuals with diabetes two to four times more likely to develop CVDs like heart attacks, strokes, and peripheral arterial disease. Key cardiovascular metrics like heart rate, blood pressure, oxygen saturation, and blood glucose levels are crucial for clinicians.^8^, ^9^, ^10^

Machine learning algorithms can be trained using these metrics, and their advantage lies in their ability to analyze a broader range of variables than traditional methods.^11^, ^12^ Abductive and inductive reasoning play complementary roles in the application of machine learning to data analysis.^13^, ^14^ Abductive reasoning involves inferring the most plausible explanation for observed data, even when the information is incomplete or noisy. In machine learning, this is analogous to the model's ability to hypothesize potential relationships or patterns that fit the data, such as identifying novel biomarkers or risk factors in cardiovascular datasets. Inductive reasoning, in contrast, involves deriving general principles from specific data observations. In machine learning, this process underpins the development of predictive models that can generalize patterns from the training dataset to unseen data. Machine learning algorithm may observe a correlation between specific biomarkers and cardiovascular risk in one dataset and use inductive reasoning to predict similar risks in new patient populations. Through abductive and inductive reasoning, machine learning reveals complex patterns in datasets, improving the identification of cardiovascular risks. Point‐of‐care (POC) devices, which offer rapid and accurate testing near the patient, combined with machine learning, promise to accelerate the development of labor‐free, intelligent diagnostics for CVDs healthcare.^15^, ^16^, ^17^, ^18^

A diagnostic test comprises two key components: signals or markers indicating a disease and mechanism for their reliable detection and measurement.^19^ In CVDs healthcare, a biomarker is a quantifiable indicator produced by biological processes, often signaling the likelihood of disease. For effective CVDs diagnosis, the biomarker must distinguish between individuals with other active diseases. Biomarkers for POC tests in CVDs need to exhibit high specificity to CVDs and be detectable at concentrations that allow quick and simple testing procedures. The challenges in timely CVDs diagnosis stem from two primary factors.^15^, ^19^, ^20^ First, the chronic nature of CVDs and low awareness levels can prevent patients from seeking timely medical attention. Barriers like restricted access to healthcare, economic and geographical challenges, and concerns about social stigma further contribute to delayed care. Second, limited diagnostic capabilities arise from under‐resourced health systems and outdated diagnostic technologies, hindering early detection and effective intervention. Patients often need multiple visits over extended periods, complicating diagnosis due to varying pathology across different patient groups, such as children and immunosuppressed individuals. To address limitations of current diagnostics in medical centers, a POC test that does not require laboratory facilities or specialized training is urgently needed.^21^, ^22^, ^23^ Key considerations for POC diagnostics include sensitivity, specificity, and predictive value, with immediate treatment initiation for positive results being crucial.^19^, ^24^, ^25^ While high sensitivity is preferred, even modest improvements over current diagnostics are acceptable if they increase accessibility. The test should be robust, affordable, and capable of using non‐invasive samples like blood, urine, or breath, ensuring convenience and safety. The Foundation for Innovative New Diagnostics (FIND) expert workshop outlined the minimum specifications for an optimal POC test, highlighting the importance of equipment‐free operation, independence from electrical power, and resilience to environmental conditions.^15^, ^19^, ^20^, ^26^

In machine learning domains, three main algorithms exist: unsupervised, supervised, and semi‐supervised learning.^27^, ^28^, ^29^ These algorithms are categorized based on the availability of labeled data. Supervised techniques use labeled data to determine feature importance by measuring its correlation with class labels.^30^, ^31^, ^32^ Unsupervised methods assess feature relevance by preserving data properties like variance or spatial structure.^33^ Although supervised techniques often yield better performance than unsupervised ones, they depend on a large volume of labeled data, which is costly and labor‐intensive to gather. In cases where labeled data is scarce, semi‐supervised feature selection is increasingly applied. Rather than relying on pre‐defined equations, machine learning models use abductive and inductive reasoning to discover hidden patterns in the data. Machine learning has been employed in cardiovascular research for years, such as in analyzing electrocardiograms (ECGs) based on fundamental AI principles.^34^, ^35^, ^36^ The resurgence of interest in machine learning is fueled by modern, scalable computing systems and algorithms capable of processing large datasets in real time.^37^, ^38^ Although the complexity of these datasets poses analytical challenges, it also enables deeper insights. The key benefit of machine learning lies in its capacity to handle intricate, multidimensional, and nonlinear interactions between variables.^39^, ^40^, ^41^ Machine learning's ability to navigate these intricate relationships is crucial for cardiovascular healthcare. Traditional methods often struggle to decode the complex links between health indicators and outcomes, a challenge that machine learning can tackle. Recent advancements in computational infrastructure, specifically optimized for machine learning algorithms, have initiated a revolutionary phase in data‐driven methodologies.^42^, ^43^, ^44^ This progress enables in‐depth analysis and reliable predictions concerning cardiovascular risks across a variety of models.^45^

In this review, we summarize emerging POC devices and machine learning models for CVDs healthcare. Timely diagnosis and continuous monitoring are essential for managing conditions such as arteriosclerosis, myocardial infarction, coronary artery disease, and hypertension. Machine learning algorithms, capable of analyzing complex data, provide improved accuracy in identifying cardiovascular risks compared to traditional methods. Integrating these machine learning algorithms with POC devices allows for rapid, precise diagnostics and more effective management of CVDs.

## 2 POINT‐OF‐CARE DEVICES FOR CARDIOVASCULAR HEALTHCARE

In low‐resource environments, conventional medical devices typically found in well‐equipped laboratories encounter numerous obstacles. These include inconsistent power supply, a lack of qualified personnel, harsh environmental conditions, and inadequate data connectivity.^15^, ^20^, ^46^, ^47^ Consequently, clinical decisions in these contexts often rely on symptomatic observations rather than diagnostic tests, which can lead to more complicated outcomes. Although several POC devices for diagnosing CVDs have been created, their use in low‐resource environments is limited by elevated costs, a lack of skilled personnel, and reliance on supplementary equipment.^48^, ^49^, ^50^

**FIGURE 1:**
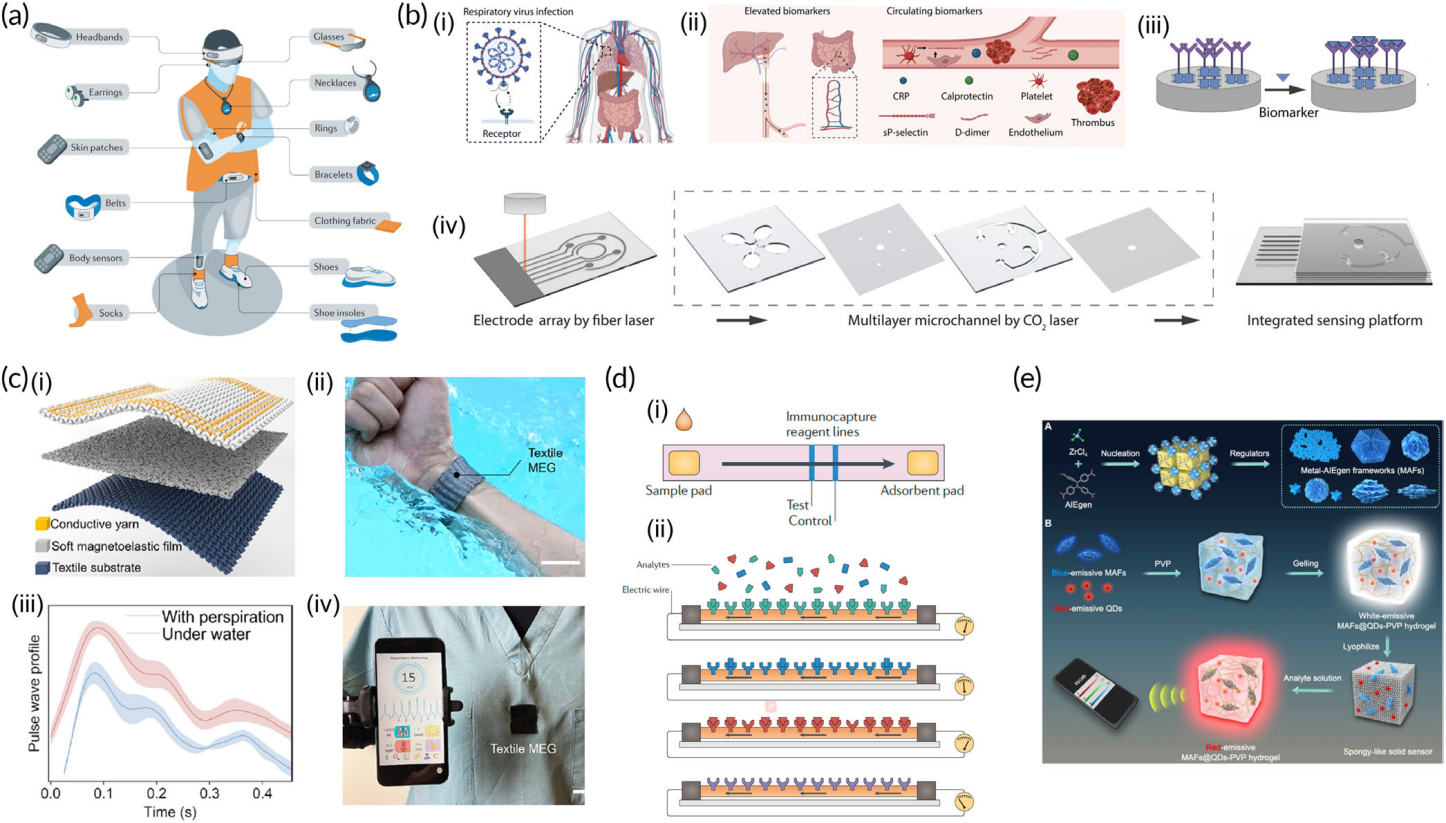
Point‐of‐care (POC) devices for cardiovascular healthcare. (a) Overview of present and emerging wearable technologies. Reproduced with permission.^45^ Copyright 2021, Springer Nature. (b) Nanoengineered multichannel immunosensors with unsupervised clustering to predict acute thrombosis. Reproduced with permission.^52^ Copyright 2024, American Association for the Advancement of Science (AAAS). (c) Magnetoelastic generator (MEG)‐based e‐textiles have been designed for advanced biomechanical sensing applications. Reproduced with permission.^15^ Copyright 2021, American Chemical Society (ACS). (d) Nanosensor technology, where silicon nanowires, arranged on microchips and coated with specific molecules, detect analytes through changes in electrical conductance, allowing for instant detection and reuse upon dissociation of the analytes. Reproduced with permission.^20^ Copyright 2011, Springer Nature. (e) Depicts the synthesis process for various metal–organic frameworks (MAFs) and the employed digital sensing strategy. Reproduced with permission.^55^ Copyright 2022, AAAS.

Biosignals, which encompass electrical, chemical, and mechanical activities, are essential for assessing health and disease, forming the foundation for creating personalized physiological profiles. Wearable technologies now exist to measure a range of biosignals (Figure 1a).^45^ Some devices can simultaneously acquire multiple biosignals, feeding data into sophisticated diagnostic and monitoring systems. The immediate aim focuses on adopting guideline‐based care, whereas the longer‐term aim is persistent physiological monitoring. Innovative sensors, such as wearable chest patches, are capable of tracking metrics like heart rate, respiration, and skin temperature. Current advancements are expanding to monitor myocardial contractility, cardiac output, and heart sounds.^5^, ^51^

Acute viral infections are a significant public health concern, with increasing evidence linking respiratory viral infections, such as COVID‐19, to clinical thrombotic events (Figure 1b).^52^ These events, including acute coronary syndromes, cerebrovascular accidents, and pulmonary embolism, highlight the prothrombotic state caused by such infections and underscore the need for effective risk prediction tools. Accurate and rapid methods for assessing thrombotic risk are critical for guiding anticoagulation prophylaxis and managing future waves of severe infections like SARS‐CoV‐2. While single biomarker offers valuable insights, predicting thrombotic events remains challenging due to the multifactorial nature of the condition, including viral strain variability and individual health factors. Single‐biomarker approaches often lack the accuracy needed, necessitating the use of integrated, multi‐biomarker strategies. To address this, Kaidong Wang et al. developed a nanoengineered multichannel immunosensor for the simultaneous detection of multiple biomarkers (Figure 1b).^52^ To construct the nanoengineered multichannel immunosensor, a polyvinyl chloride substrate was made electrically conductive by spraying it with acid‐treated carbon nanotubes (CNTs). A fiber laser was then used to etch a predesigned pattern onto the CNTs substrate, forming multiplexed electrodes. Detection and wash chambers were engraved using a CO_2_ laser. Gold nanoparticles (Au NPs) were electrochemically deposited onto the electrodes and functionalized with aptamers for CRP, sP‐selectin, and D‐dimer via Au‐S bonding. Calprotectin antibodies were immobilized using EDC/NHS chemistry. The resulting impedance changes at the electrode interface facilitated effective biomarker detection (Figure 1b). Coupled with unsupervised clustering algorithms, this approach stratifies patients by thrombotic risk, enabling personalized assessments and improved management of complications like stroke and pulmonary embolism.^52^

The magnetoelastic effect, typically observed in rigid metals and alloys, has recently been discovered in soft materials, unlocking new possibilities for biomechanical‐to‐electrical energy conversion and soft bioelectronics. The Chen group developed a soft magnetoelastic generator (MEG) by embedding NdFeB‐based micromagnets in a polymer matrix, which align in a wavy chain‐like structure under a magnetic impulse, retaining significant magnetization. MEGs, being waterproof, can function as self‐powered biosensors for monitoring physiological signals like pulse waves and respiratory rate, even in sweat or underwater (Figure 1c).^15^ A textile MEG was created by sewing a soft magnetoelastic film with a textile coil, allowing comfortable integration into clothing for real‐time health monitoring. This technology demonstrated high sensitivity, quick response time, and durability, withstanding prolonged exposure to water and perspiration. A customized mobile application enables real‐time analysis and wireless transmission of health data for clinical diagnostics. Additionally, a new woven e‐textile MEG offers improved air permeability and comfort, with microbubbles inside the fibers enhancing softness and reducing density. These textile biomechanical sensors provide continuous, high‐quality health monitoring data for disease diagnosis. Integrating these sensors with the Internet of Things (IoT) could revolutionize healthcare by enabling wireless transmission of patient data for wearable POC systems.^53^, ^54^

Lateral‐flow immunochromatography is a widely used technology in POC tests, commonly referred to as dipsticks (Figure 1d).^20^ These devices employ antibody–antigen interactions paired with enzymes that produce a noticeable color shift upon contact with a specific substrate, delivering quick and easy‐to‐interpret results within minutes. The accuracy of these diagnostic tests relies on the concentration and presence of the target analyte in samples. However, their ability to detect multiple targets is limited, which can pose challenges for accurately diagnosing diseases, necessitating more advanced microfluidic devices. Nanotechnology, which manipulates structures at the molecular level, can improve detection by increasing the number of reactive sites, allowing for simultaneous detection of multiple markers. By incorporating nanotechnology into conventional biomarker detection methods like lateral‐flow assays, sensitivity can be improved while maintaining high specificity (Figure 1d).^20^ Coating different materials with nanoparticles enables the creation of single‐use devices that generate detectable optical or electrical signals, allowing for real‐time analysis. Though still in the early stages for disease diagnosis, nanotechnology shows promise for improving detection and molecular analysis, although large‐scale manufacturability and cost remain significant challenges.

POC biochemical sensors are extensively used in clinical diagnostics, but they often face challenges related to low sensitivity. To address this issue, metal‐AIEgen frameworks (MAFs) were developed by incorporating emission luminogens (AIEgens) into metal–organic frameworks (MOFs), achieving a high quantum yield (~99.9%) (Figure 1e).^55^, ^56^, ^57^, ^58^, ^59^ These MAFs significantly enhance the sensitivity of hydrogel‐based POC digital sensors and lateral flow immunoassays (LFIA) by 102‐ to 103‐fold. MAFs have a high affinity for proteins, enabling efficient antibody labeling without the need for stabilizing polymers or covalent linking agents, making them ideal for POC applications. By integrating MAFs with blue‐emissive mesoporous and red‐emissive quantum dots, a white‐emissive POC hydrogel digital sensor (HDS) provided a white‐to‐red fluorescence signal for easy visual detection and quantitative analysis via smartphone‐based RGB analysis. The use of MAFs in LFIAs has demonstrated remarkable sensitivity, selectivity, and reliability for clinical diagnostics, offering a promising solution to enhance the performance of POC sensors. This advancement in MAF‐based POC sensors addresses the critical need for sensitive, rapid, and convenient diagnostic tools in healthcare.

## 3 MACHINE LEARNING ALGORITHMS IN CARDIOVASCULAR HEALTHCARE

Herein, common machine learning concepts such as backpropagation,^60^ maximum margin classifiers,^61^ Fisher criterion,^62^ and Laplacian score^63^ are essential for understanding the field. Backpropagation, a fundamental algorithm for neural network training, computes the gradient of the loss function concerning the model's weights using the chain rule of calculus. This allows for iterative weight adjustments to minimize prediction errors. The maximum margin classifier, commonly linked to Support Vector Machines (SVMs), establishes a decision boundary that maximizes the separation between classes, improving generalization. The Fisher criterion, used in linear discriminant analysis (LDA), evaluates the separability of classes by maximizing the variance between classes relative to the variance within them. Meanwhile, the Laplacian score ranks features based on their ability to maintain local data structure, making it particularly useful in semi‐supervised learning.

The process of model training and evaluation is also critical to understand machine learning.^31^, ^32^, ^64^ The dataset is typically divided into three subsets: a training set for learning patterns, a validation set for hyperparameter tuning and preventing overfitting, and a test set for assessing model performance. Model training begins with initializing parameters, followed by a forward pass to generate predictions, a loss calculation to measure error, and backpropagation to compute gradients. The parameters are optimized using algorithms like Stochastic Gradient Descent or Adam to reduce error iteratively. Validation involves periodic checks to ensure the model generalizes well, adjusting for overfitting when needed. Finally, testing evaluates the model's accuracy, precision, recall, and other metrics, providing insight into its ability to handle unseen data.

**FIGURE 2:**
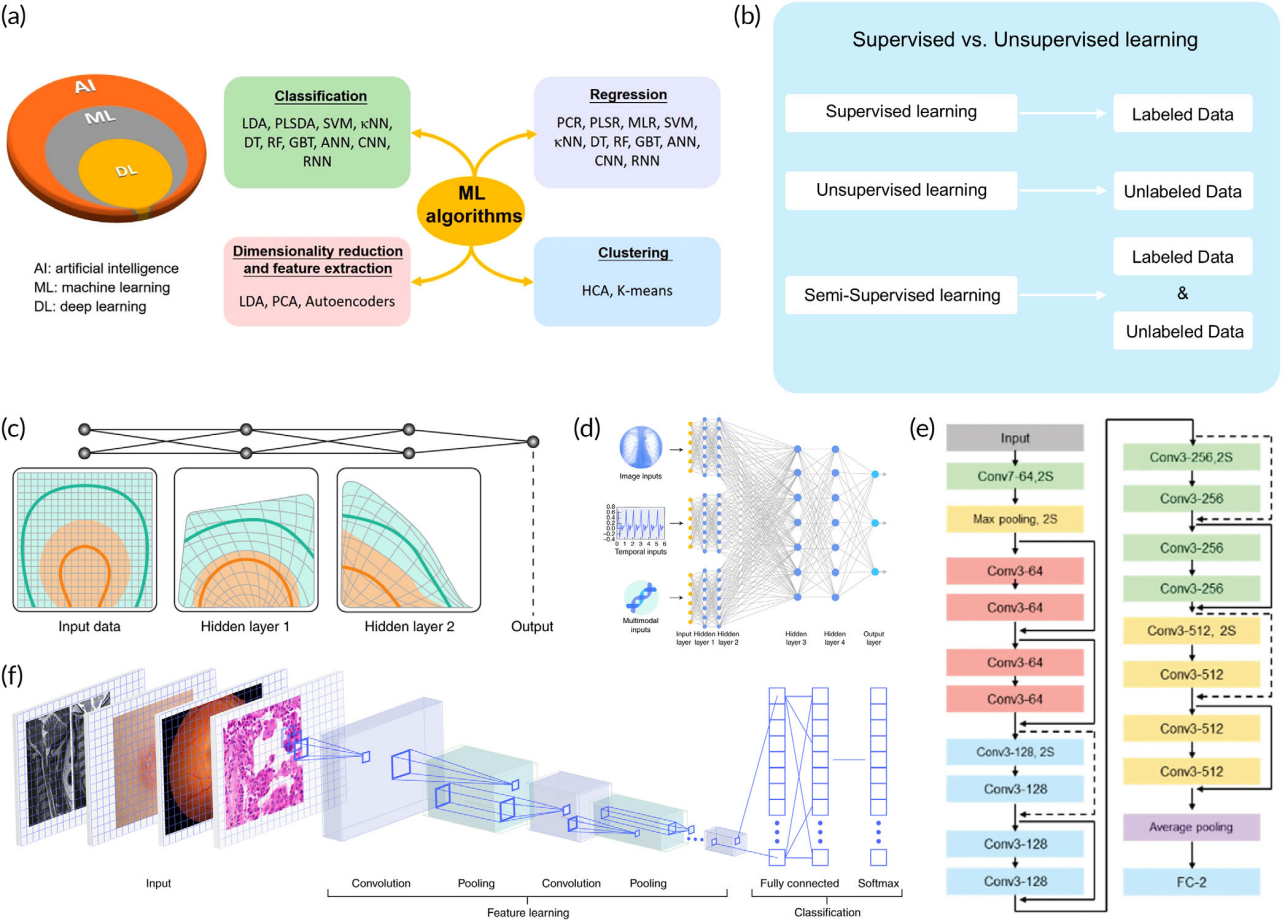
Overview of machine learning classifications and algorithms. (a) Key machine learning algorithms. Reproduced with permission.^65^ Copyright 2020, American Chemical Society (ACS). (b) Comparative analysis of supervised, unsupervised, and semi‐supervised learning paradigms. (c) Visualization of input data: Hidden layer 1, hidden layer 2, and output. Reproduced with permission.^74^ Copyright 2019, Springer Nature. (d) An example of a large‐scale neural network that takes various data types as input, learning effective feature representations in its lower‐level towers for each data type. Reproduced with permission.^74^ Copyright 2019, Springer Nature. (e) Illustration of the architecture of ResNet‐18 model. (f) CNNs in medical applications. Reproduced with permission.^74^ Copyright 2019, Springer Nature.

Figure 2a illustrates the various types of machine learning classifications and algorithms.^65^ In general terms, machine learning is generally categorized into three primary types: supervised learning, unsupervised learning, and semi‐supervised learning (Figure 2b). These categories are defined by the presence or absence of labeled data. This section provides an in‐depth examination of the algorithms that fall under each machine learning category.

### 3.1 Supervised learning

In the realm of supervised learning (SL), a training set is denoted by *X* with elements ranging from *x*_1_ to *x*_n_.^66^ The primary objective is to formulate a function, *f*, that maps inputs from *X* to their corresponding outputs in *Y*. This function, selected from a pre‐determined set *F*, enables the prediction of an output label, *y*, for any given input sample, *x*, within *X*. Depending on the characteristics of the output domain *Y*, SL tasks are divided into classification and regression. Classification pertains to scenarios in which the output label, *Y*, is discrete, whereas regression involves continuous output labels. Each label in *Y* is categorized as a class. Binary classification arises when there are only two possible outputs, while multi‐class classification refers to scenarios with more than two possible outputs.^67^ For the accurate modeling of function *f*, a substantial number of samples are generally required. A limitation of SL is that if the sample size is inadequate, the predefined function might exhibit suboptimal performance on previously unseen samples. Another challenge is the extensive manual effort and time required to label vast quantities of data.^68^

SL involves the conceptualization of a function based on known input–output pair samples. It encompasses methods like support vector machines (SVM), decision trees (DTs), random forests (RFs), various regression frameworks, multilayer perceptron (MLP), convolutional neural networks (CNNs), and recent advances in DL architectures.^31^, ^64^, ^69^

#### 3.1.1 Multilayer perceptron

The multilayer perceptron (MLP) is a computational framework that draws from the architecture of biological neural systems.^70^ It is adept at autonomously optimizing model parameters using data, typically through a process known as backpropagation. A typical MLP consists of an input layer, one or more hidden layers, and an output layer. Variations in MLP models stem from adjustments in architecture, input configurations, and layer structures.

#### 3.1.2 Support vector machine

Support vector machine (SVM) is a powerful machine learning model that effectively addresses classification tasks.^71^ At its core, SVM operates as a maximum margin classifier, focusing on enlarging the gap that separates different classes. While the traditional maximum margin approach adopts a hard margin for differentiation, it remains vulnerable to outliers. To counteract this, SVM implements a soft margin classification strategy. This mechanism aims to discern the optimal soft margin, optimizing classification scores.

#### 3.1.3 Regression models

Regression analysis is used to forecast a dependent variable by defining a function that represents the relationship between it and one or more independent variables.^72^ Typically, this method is utilized to forecast target values that are not present within the training dataset.

#### 3.1.4 Decision trees and random forests

Decision trees constitute a form of supervised machine learning that iteratively segregates data based on specific criteria, ultimately leading to a structure comprising decision nodes and leaves. Random forests (RFs), on the other hand, represent an ensemble method that amalgamates multiple DTs. Within RFs, subsets of observations and attributes are randomly selected to construct distinct trees, resulting in a heterogeneous and uncorrelated set of trees.^73^

#### 3.1.5 Deep learning

Deep learning, an advanced area within machine learning, leverages multi‐layered neural networks (usually three layers or more) and is highly effective in handling complex healthcare datasets. The neural networks, capable of image and voice recognition, optimize model parameters for medical diagnosis and classification, making it a powerful tool in cardiovascular healthcare. Figure 2c–f illustrates the applications of deep learning methods in healthcare.^74^, ^75^, ^76^ In deep learning, machines are fed raw data directly, thereby enabling them to independently establish the required patterns for recognition. This identification is accomplished via multiple layers, each adding a level of abstraction to the representation through numerous basic, nonlinear procedures. As data progresses through these layers, its structure undergoes successive transformations, eventually making individual data points discernible (Figure 2c).^74^ As shown in Figure 2d, the deep learning systems are versatile, capable of processing diverse data types, making them especially suitable for varied healthcare data sets.^74^ Training deeper neural networks poses several challenges. To address this, a residual learning approach has been employed, allowing for the training of much deeper networks than traditionally used (Figure 2e).^77^ This methodology, known as Resnet, has demonstrated exceptional performance in feature extraction, as evidenced in Figure 2e.^2^, ^77^ This approach shifts the focus towards learning residual functions for the layer inputs. As shown in Figure 2f, medical imagery stands to gain significantly from the latest developments in image categorization and object recognition.^78^, ^79^ Research across various fields, including dermatology, radiology, ophthalmology, and pathology, has yielded promising results in complex diagnostic tasks.^80^, ^81^ Deep learning platforms have the potential to augment the capabilities of medical professionals by offering additional insights and highlighting areas requiring attention in images.^82^, ^83^

These neural networks are designed to mimic brain function, allowing them to learn and adapt from vast datasets.^84^ Deep learning powers a range of AI applications that enable both analytical and physical tasks to be performed independently.^76^ Many modern conveniences, including automated customer support and sophisticated medical diagnostics, are powered by deep learning technology. At the core of deep learning is the artificial neural network. It consists of interconnected nodes that are analogous to neurons and is organized into three primary layers: input, hidden, and output layers. Deep learning models necessitate extensive training using large datasets. In deep learning, the term “deep” indicates the multiple layers data passes through for processing and transformation. Additional layers allow for increased complexity. Deep learning has diverse applications, such as image and speech recognition, medical diagnostics, fraud detection in finance, and multiple types of classification tasks. Especially during the training phase, deep learning models are resource‐intensive. This has prompted the creation of specialized hardware for efficient computation.^85^, ^86^ Numerous frameworks and libraries, including but not limited to TensorFlow and PyTorch, are available to aid in the advancement of deep learning models.^87^ In the last decade, deep learning has advanced rapidly, thanks to the increasing availability of large datasets known as big data, advancements in neural network algorithms, and the advent of powerful GPUs. With ongoing advancements, deep learning is poised to drive further innovation across multiple fields.

### 3.2 Unsupervised learning

Unsupervised learning involves datasets that lack supervised information, represented as a set *X* containing elements from *x*_1_ to *x*_n_.^88^ Without prior guidance, identifying relationships between these samples can be intricate. A common UL task is clustering, which aims to categorize the *n* samples into various groups.^89^ Ideally, similar samples fall into the same category while different ones are allocated to separate groups. The cluster count is typically set by the user. For an in‐depth understanding of the clustering method, one can consult certain references. However, the absence of labeled information in unsupervised learning can sometimes hinder the prediction of expected outcomes for specific input data.

**FIGURE 3:**
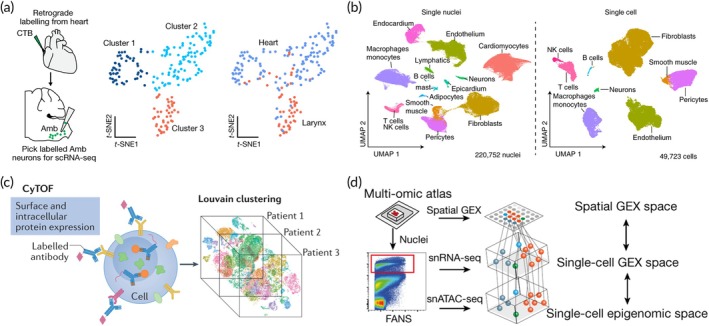
Unsupervised learning in cardiovascular healthcare. (a) scRNA‐seq of Amb^Cardiac^ neurons identifies a genetic signature of brainstem parasympathetic neurons. Reproduced with permission.^91^ Copyright 2022, Springer Nature. (b) Using the unsupervised uniform manifold approximation and projection (UMAP) technique to cluster 220,752 nuclei and 49,723 cells. Reproduced with permission.^93^ Copyright 2022, Springer Nature. (c) CyTOF can be employed to examine the diverse cellular categories and prevalence of immune cells among patients, utilizing impartial methods like Louvain clustering. Reproduced with permission.^95^ Copyright 2022, Springer Nature. (d) A 10‐μm cryo‐section was made to extract nuclei from the tissue adjacent to it, followed by fluorescence‐activated nuclei sorting (FANS) for single‐nucleus RNA sequencing (snRNA‐seq) and single‐nucleus ATAC sequencing (snATAC‐seq). Reproduced with permission.^97^ Copyright 2022, Springer Nature.

Unsupervised learning employs algorithms that identify underlying patterns and insights in unlabeled datasets. Typical applications in this area include clustering, association analysis, and principal component analysis (PCA).^90^ There are many studies on unsupervised learning in cardiovascular healthcare, as depicted in Figure 3. Avin Veerakumar and colleagues utilized methods such as retrograde neuronal tracing, single‐cell RNA sequencing, optogenetics, and physiological assessments to investigate the cardiac parasympathetic regulation circuit. They discovered that the brainstem nucleus ambiguous (Amb) contains neurons affecting the heart, which can be categorized into two unique subtypes in terms of their molecular structure, anatomy, and function (Figure 3a).^91^ Heart failure is a leading contributor to global morbidity and mortality.^92^ The emergence of single‐cell transcriptomics has transformed our understanding of cellular architecture and associated gene expression profiles. Andrew L. Koenig and colleagues applied the unsupervised Uniform Manifold Approximation and Projection (UMAP) method to cluster a dataset comprising 220,752 nuclei and 49,723 cells (Figure 3b).^93^ This investigation contributes to investigating distinct transcriptional markers linked with age and heart failure, thereby providing insights into the emergence of specific cell states related to the disease. Inflammation plays a crucial role throughout all phases of atherosclerosis and remains a significant ongoing cardiovascular risk, even in patients who receive optimal treatment.^94^ As shown in Figure 3c, CyTOF can be employed to examine the diverse cellular categories and prevalence of immune cells among patients, using impartial methods like Louvain clustering.^95^ Heart attacks rank among the leading global causes of mortality.^96^ While progress has been made in immediate treatments, our limited insights into the remodeling processes have limited the success of interventions aimed at reducing long‐term mortality. From each heart sample, 0‐μm thick cryo‐sections were obtained. Nuclei were subsequently extracted from adjacent tissue (Figure 3d).^97^

#### 3.2.1 Self‐organizing maps

Self‐organizing maps (SOM) represents an unsupervised neural network approach.^98^ It is adept at clustering high‐dimensional datasets and converting these high‐dimensional attributes into two‐dimensional representations, ensuring the preservation of symmetrical relationships among the samples.

#### 3.2.2 Clustering algorithms

Clustering represents an unsupervised machine learning technique that intuitively categorizes data based on inherent similarities, typically defined by a pre‐determined distance metric.^99^, ^100^ This technique has been crucial in uncovering unanticipated associations between parameters and optimizing various machine learning strategies.

### 3.3 Semi‐supervised learning

Semi‐supervised learning was introduced to leverage the strengths of both unsupervised learning and supervised learning while mitigating their shortcomings.^101^, ^102^ Positioned between the two, semi‐supervised learning amalgamates features of both. In this learning paradigm, dataset *X* contains a mixture of a few labeled samples and most unlabeled samples. Specifically, *X*_l_ denotes the labeled subset, while *X*_u_ signifies the unlabeled samples. Commonly, the number of labeled samples is much smaller than the unlabeled ones. The goal is to predict labels for the unlabeled samples, utilizing the relationships gleaned from the available labeled data. Various assumptions guide semi‐supervised learning algorithms to facilitate the transfer of label information to the unlabeled samples. These include the smoothness assumption, which implies that samples situated closely in a high‐density region likely have similar outputs. The cluster assumption suggests that samples grouped within the same cluster are likely part of the same class, while the manifold assumption posits that samples near each other in high‐dimensional space will stay close when projected onto a lower‐dimensional manifold. Manifold regularization effectively handles previously unseen data points.

Semi‐supervised learning employs both labeled and unlabeled datasets.^103^ Within this learning framework, certain guiding principles, such as the cluster and manifold assumptions, are essential. The clustering principle implies that samples within the same cluster tend to belong to the same class, while the manifold principle suggests that high‐dimensional data often lies on an underlying lower‐dimensional structure. Various approaches to semi‐supervised learning exist. Semi‐supervised filter methods are computationally efficient but frequently ignore dependencies between features. In cases of high feature correlation, embedded methods or sparsity‐based filter techniques may be more effective. These methods consider feature interdependencies but require robust iterative algorithms to handle the non‐smooth objective functions involved.

#### 3.3.1 Generative models

Semi‐supervised generative models operate on the principle that they use a formula, represented as *p*(*x*, *y*) which is derived from the amalgamation of *p*(*y*) and *p*(*x*|*y*).^104^, ^105^ Here, *p*(*x*|*y*) signifies a unique distribution. When an abundance of unlabeled data is present, these mixture components become distinguishable. Consequently, to adequately define this mixture distribution, only a single labeled instance for each component is required.

#### 3.3.2 Self‐training

Self‐training starts by initially training a classifier using labeled data.^105^, ^106^ The classifier is used to assign labels to the unlabeled data. Next, a subset of this unlabeled data, where the classifier shows the highest confidence in its predictions, is chosen and added to the training set along with the predicted labels. The classifier is then updated with this augmented dataset, and the process is iterated. Essentially, self‐training enables the classifier to enhance its accuracy by learning from its predictions.

#### 3.3.3 Co‐training

Co‐training utilizes two distinctive classifiers that operate on two separate sets.^107^, ^108^ After training each classifier on its respective feature set, they predict labels for the unlabeled data. The predictions with the highest confidence from one classifier are used as labeled training data for the other, and this iterative process continues in cycles.

#### 3.3.4 Semi‐supervised filter feature selection methods

The feature selection is categorized into six distinct domains: approaches anchored in spectral graph theory and the cluster assumption; techniques focusing on the Laplacian score; methods that are dependent on pairwise constraints; strategies that use the Fisher criterion; techniques leveraging sparse models.^109^, ^110^

## 4 ADVANCED STRATEGIES TOWARDS CARDIOVASCULAR HEALTHCARE

The advancements of POC devices leveraging machine learning have led to innovative strategies in cardiovascular healthcare.^111^, ^112^ These technological improvements allow for more frequent detection of irregularities in heart health metrics, thereby paving the way for more proficient, automated evaluations. Enhanced diagnostic methods can improve the accuracy and broader applicability of results obtained from these devices.

### 4.1 Wearable devices for continuous monitoring

**FIGURE 4:**
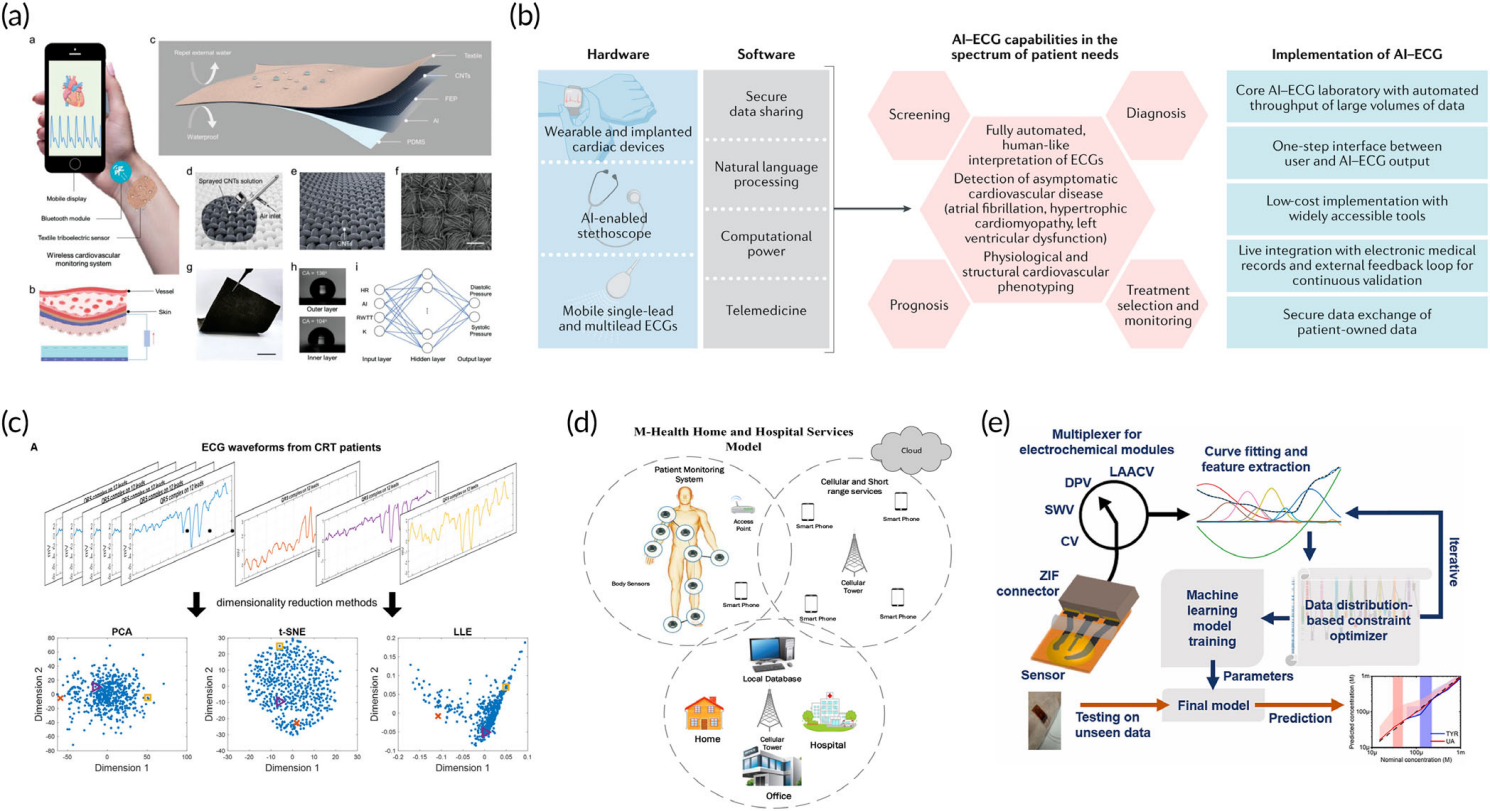
Machine learning‐assisted point‐of‐care (POC) devices for cardiovascular healthcare. (a) Design of a textile cardiovascular monitoring system, featuring a self‐powered triboelectric sensor that wirelessly transmits cardiovascular signals to a cellphone. Reproduced with permission.^114^ Copyright 2021, Wiley‐VCH. (b) The integration of artificial intelligence (AI) in clinical practice is being facilitated by modern electrocardiogram (ECG) technologies, which enable the storage, transfer, processing, and analysis of large volumes of digital data to meet diverse patient needs. Reproduced with permission.^117^ Copyright 2021, Springer Nature. (c) Unsupervised machine learning is used for dimensionality reduction and k‐means clustering to identify two subgroups of cardiac resynchronization therapy (CRT) patients based on ECG QRS complex waveforms. Reproduced with permission.^118^ Copyright 2020, American Heart Association (AHA). (d) Emerging Mobile Health (M‐Health) systems, leveraging advanced technologies such as deep learning, artificial intelligence, cloud computing, and big models. Reproduced with permission.^124^ Copyright 2020, Elsevier. (e) Machine learning‐powered multimodal analytical device utilizing electrodeposited molybdenum polysulfide on laser‐induced graphene for multiplexed detection of tyrosine and uric acid in sweat and saliva. Reproduced with permission.^130^ Copyright 2022, Elsevier.

Developing wearable bioelectronics that can reliably monitor pulse waves amidst body movement and perspiration remains a significant challenge.^32^, ^113^ Figure 4a introduces a low‐cost, lightweight, and durable textile triboelectric sensor designed to convert subtle skin deformations from arterial pulsations into electrical signals for continuous pulse monitoring in ambulatory settings.^114^, ^115^ Enhanced by machine learning algorithms, this sensor can accurately measure systolic and diastolic pressure, validated against a commercial blood pressure cuff. A customized mobile application enables one‐click data sharing and real‐time cardiovascular diagnosis. The sensor's design includes waterproof and conformable properties, with an outer textile layer that protects against noise and moisture and an inner PDMS layer for biocompatibility and waterproofing. The conductive network, formed by spray‐coating CNTs onto a cotton textile, ensures low resistance and flexibility.^115^, ^116^ This system represents a shift towards personalized, predictive, and preventative healthcare, seamlessly integrating with daily life and the IoT to provide continuous and precise health monitoring.

Textiles have played a crucial role in human civilization, evolving from natural materials like silk and cotton to advanced synthetic options such as peptides and polyester. These modern materials can now be engineered to be biocompatible, biodegradable, and even bioabsorbable, making them ideal for on‐body healthcare applications. By integrating biomedical electronics, electronic textiles (e‐textiles) have been developed to offer POC diagnostics and therapeutic functions while ensuring comfort, breathability, and durability. E‐textiles can monitor vital signals through various sensors and provide therapeutic support.

Moreover, they can incorporate protective features and leverage wireless technologies like Near Field Communication (NFC) and Bluetooth, creating a closed‐loop system for continuous monitoring and treatment. With the advent of 5G, enhanced connectivity and telemedicine will enable more precise, comprehensive, and personalized medical care. The integration of wearable devices with machine learning analysis transforms them into efficient POC platforms, capable of continuous monitoring and real‐time treatment directly on the patient. This synergy between wearable sensors, 5G, and the IoT will revolutionize healthcare by enabling real‐time data transmission and processing, leading to the development of intelligent POC textile platforms that shape the future of personalized healthcare in the IoT era.

### 4.2 Implementation of machine learning ECGs

The application of machine learning to ECGs analysis is transforming cardiovascular medicine by providing rapid, human‐like interpretations and identifying patterns that are imperceptible to humans (Figure 4b).^117^ Cutting‐edge machine learning methods, especially deep‐learning convolutional neural networks, have transformed ECGs into valuable non‐invasive biomarkers for detecting conditions like left ventricular dysfunction, silent atrial fibrillation, and hypertrophic cardiomyopathy. Extensive datasets of digital ECGs connected to clinical information have supported the creation of machine learning models, which are now undergoing clinical trials to confirm the real‐world impact of these algorithms in primary care settings. Machine learning‐enhanced ECGs have the potential to greatly improve screening, diagnosis, prognostication, and personalized treatment by leveraging the simplicity and reliability of ECGs data collection. The integration of machine learning‐ECGs with electronic health records and mobile technologies enhances clinical decision‐making, patient monitoring, and data sharing, ultimately advancing personalized healthcare. Current research is actively investigating the use of machine learning algorithms for detecting atrial fibrillation and preventing strokes. As these technologies evolve, they are poised to revolutionize clinical practice, improve patient outcomes, and optimize healthcare delivery.

Unsupervised learning is less developed than supervised machine learning but holds significant potential for identifying natural patterns within data without using predefined labels. Unlike supervised learning, which trains models to predict outcomes, unsupervised learning focuses on uncovering inherent structures within the data. In one example, applying unsupervised learning to clinical data and echocardiographic metrics during the trial uncovered distinct heart failure patient phenotypes, each showing varied responses to cardiac resynchronization therapy (CRT) (Figure 4c).^118^ Cluster analysis, a common unsupervised technique, groups similar data points, with hierarchical agglomerative clustering creating larger clusters from smaller ones and often visualized using dendrograms and heat maps. Alternatively, k‐means clustering partitions data into a specified number of clusters based on proximity to cluster centroids. Dimensionality reduction methods, which condense complex, high‐dimensional data into fewer dimensions while maintaining its structural integrity, can aid in effective clustering.

Advanced machine learning algorithms have shown proficiency in detecting specific waveforms in ECG analysis.^2^, ^5^, ^119^, ^120^ Leveraging these technological innovations enables precise calculation of essential clinical metrics. Emerging models excel in identifying not just ST‐segment fluctuations but also common rhythm disorders. Central to this advancement are well‐established machine learning techniques, such CNNs, SVMs, and hidden Markov models (HMMs). Recent research, particularly in signal processing and wavelet analysis, has examined numerous approaches for ECG preprocessing, feature extraction, and classification. For instance, Zhao et al. applied wavelet transform alongside autoregressive modeling to extract feature vectors from ECG traces. These vectors enabled classification of the traces into five common arrhythmias, achieving high accuracy rates on the MIT‐BIH dataset.^121^ Afsar et al. proposed a neural network that utilized wavelet‐transformed ECG signals to detect ST‐changes, which showed commendable sensitivity and predictive value.^122^ The Stanford Group made significant strides by employing a 34‐layer CNNs, surpassing even certified cardiologists in diagnosing a variety of arrhythmias.^122^ Prompt identification of individuals at risk for sudden cardiac death (SCD), particularly those with malignant ventricular arrhythmias, is paramount for timely medical intervention and improved survival rates.^123^ One research study introduced an innovative approach for the precise prediction of SCD, leveraging specific arrhythmic markers. These metrics are derived from the QRS complex waves and T‐wave of the ECG signals.

### 4.3 Mobile health systems (M‐Health)

The healthcare industry encounters obstacles in diagnosing diseases and delivering cost‐efficient services, requiring prompt treatment informed by patient data, lifestyle factors, and molecular characteristics. Technologies such as Electronic Health Records (EHR), Mobile Health (M‐Health), and Electronic Medicine (E‐Medicine) are used to tackle these challenges; however, issues in data collection, integration, and informed decision‐making remain prevalent. (Figure 4d).^124^ M‐Health technologies, models, and applications, proposed a secure, cloud‐based Android architecture to enhance data security and communication. The system also introduced machine learning techniques for predictive analysis, aimed at improving the efficiency of current healthcare systems. Given that mobile monitoring devices are generally patient‐centric, the data collected are usually directly communicated to the end‐users (Figure 4d).^45^ Endomyocardial biopsy (EMB) remains the standard procedure for identifying allograft rejections post‐heart transplant.^125^ Jana Lipkova et al. introduced a robust system, built upon deep learning, tailored for the comprehensive analysis of large‐scale EMB whole‐slide images. This system excels at pinpointing, categorizing, and ranking allograft rejection.^126^ The team validated the effectiveness of their models using a large dataset from the United States, as well as supplementary test groups from Turkey and Switzerland. This ensured a broad range of patient demographics, sample processing methods, and slide scanning techniques. When compared to traditional methods in a reader study, the AI system not only achieved comparable efficacy but also reduced discrepancies in readings and accelerated the assessment process. Such comprehensive approaches to cardiac allograft rejection evaluation pave the way for subsequent clinical trials, aimed at ascertaining the effectiveness of AI‐based EMB analysis in improving the success rates of heart transplants. This technique analyzed data, aiming to forecast the potential of readmission to the hospital. It assessed various physiological indicators including ECG, heart rhythm, respiration rate, body temperature, movement intensity, and posture among 100 HF patients. The system was able to anticipate forthcoming HF hospital admissions with a sensitivity of up to 88% and a specificity of 85%. These results are comparable to those obtained using embedded devices. Ongoing research aims to determine whether this method can proactively lower the rate of HF readmissions.

### 4.4 Electrochemical sensors for circulating biomarkers detection

The accuracy and efficiency of diagnostics are significantly enhanced through multiplexed biomarker analysis, which conserves resources and handles limited sample volumes effectively. Traditional multiplexing techniques such as Enzyme‐Linked Immunosorbent Assay (ELISA) are effective but need bulky lab‐based devices.^127^, ^128^ Electrochemical sensors, ideal for POC technology, offer portable and cost‐effective solutions for monitoring analytes, particularly redox‐active small molecules.^129^ Machine learning has been leveraged to address the challenges of overlapping redox peaks and device variability in electrochemical sensing, enabling precise multiplexed detection.^130^, ^131^ Figure 4e presents a machine learning‐enhanced equipment that employs advanced nanomaterial to detect tyrosine and uric acid.^130^, ^132^, ^133^ The platform automates electrochemical data collection and processing, enhancing detection accuracy and sensitivity.

Deepti Sharma et al. developed a 3D carbon‐based electrochemical immunosensor featuring suspended mesh and substrate‐bound interdigitated array nanoelectrodes, fabricated using the cost‐effective and scalable carbon microelectromechanical systems technology. The 3D mesh architecture and selective surface modification enabled efficient redox cycling, amplifying the signal by ~25 times and achieving a linear detection range of 0.001–100 ng/mL for cardiac myoglobin (cMyo). This immunosensor demonstrated high sensitivity, detecting cMyo at concentrations as low as ~0.4 pg/mL in both phosphate‐buffered saline and human serum, even in the presence of interfering species.^134^ Su Ryon Shin et al. developed a scalable, low‐cost microfluidic aptamer‐based electrochemical biosensor for continuous, noninvasive monitoring of drug responses by detecting trace amounts of Creatine Kinase‐MB (CK‐MB) biomarkers secreted by cardiac organoids, addressing the limitations of traditional biosensing techniques. The biosensor demonstrated high sensitivity, selectivity, and stability when integrated with a heart‐on‐a‐chip platform, effectively monitoring drug‐induced cardiac damage in a dose‐dependent manner.^135^ Monitoring cardiomyocyte‐secreted biomarkers is crucial for assessing myocardial injury caused by anticancer drugs, with cardiac troponin I (cTnI) serving as the gold standard biomarker. An electrochemical aptasensor was developed for cTnI detection, leveraging lanthanide europium metal–organic frameworks (Eu‐MOFs) and a DNAzyme‐based signal amplification strategy. The sensor achieved a detection limit of 0.17 pg/mL with a linear range of 0.5 pg/mL to 15 ng/mL and successfully measured cTnI levels in real samples, demonstrating significant potential for evaluating drug‐induced MI in clinical applications.^136^ Multiplexed detection of protein biomarkers enables early diagnosis and effective treatment of CVDs. A flexible electrochemical biosensor with vertically oriented zinc oxide nanostructures was developed for simultaneous detection of cTnI and Troponin‐T (cTnT) in a POC format. The biosensor demonstrated high sensitivity (detection limit of 1 pg/mL) and selectivity for both biomarkers in human serum, utilizing electrochemical impedance spectroscopy and Mott‐Schottky analysis for characterization.^137^ Suchanat Boonkaew et al. reported an electrochemical paper‐based analytical device was developed for simultaneous detection of three key CVD biomarkers including CRP, cTnI, and procalcitonin (PCT) using a label‐free immunoassay. The ePAD, featuring graphene oxide‐modified electrodes and square wave voltammetry for detection, demonstrated excellent sensitivity, linearity, and reproducibility, successfully quantifying the biomarkers in serum samples with promising potential for point‐of‐care applications.^138^

## 5 OUTLOOK

**FIGURE 5:**
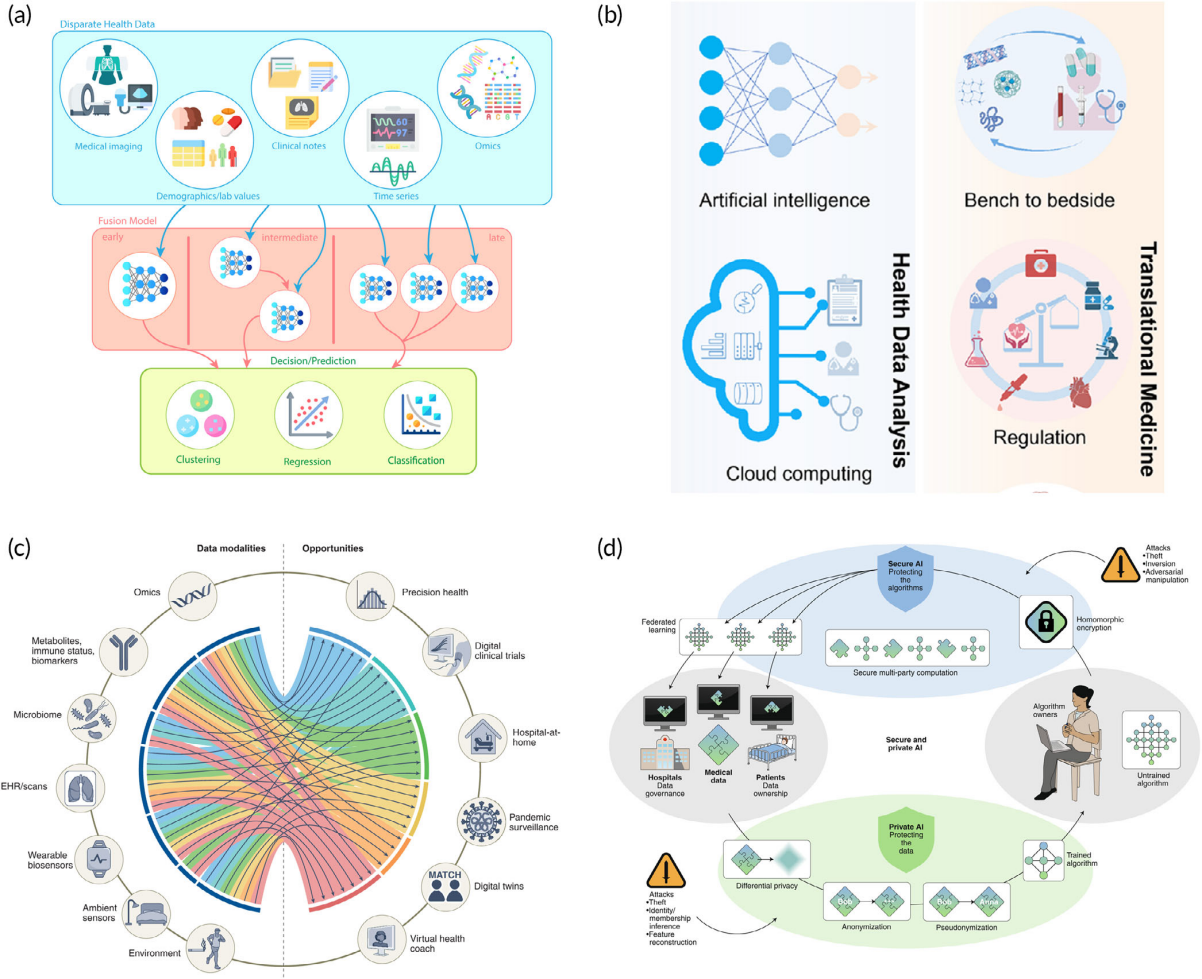
Outlook of POC devices with machine learning for cardiovascular healthcare. (a) The figure outlines a progression from early, to intermediate, to late fusion stages. This shows the flow of data from a centralized information repository, through the computational framework, and culminating in the analytical results. Reproduced with permission.^139^ Copyright 2022, Springer Nature. (b) Health data analysis enables real‐time tracking of individual health and provide preemptive medical advice, while multidisciplinary collaboration on interoperability, standards, and regulations will help bridge the gap between laboratory research and clinical practice, advancing translational research. Reproduced with permission.^15^ Copyright 2021, American Chemical Society (ACS). (c) Various types of data and their potential roles in multimodal biomedical AI.^140^ Copyright 2022, Springer Nature. (d) Schematic illustration showcasing the connections and interplay among data, algorithms, participants, and methods within the sphere of secure and confidential AI. Reproduced with permission.^163^ Copyright 2020, Springer Nature.

As illustrated in Figure 5a, data integration can be approached through various methods.^139^ There are three primary modes of fusion: early, intermediate, and late. Fusion refers to the integration of data from multiple sources or modalities to improve analytical outcomes (e.g., combining sensor outputs or patient data for tailored healthcare solutions).^140^, ^141^ Early fusion is characterized by all data being fed directly into a single model. Intermediate fusion involves a process where the outputs of one model serve as inputs for the following model. Finally, in late fusion, distinct data types are individually modeled, and the results are subsequently combined, typically through techniques like ensembling or voting. Deep neural networks, a subset of machine learning methods, are particularly effective in classifying a wide range of inputs, especially when conventional rule‐making is challenging.^142^ The parameters of this framework can be tailored to meet patient needs and adapt to various sensor types.^56^, ^57^, ^58^, ^59^, ^143^, ^144^, ^145^ Deep learning models can process large‐scale, heterogeneous datasets, including imaging, genomic, and biosensor data, which are critical for understanding complex cardiovascular conditions. Machine learning advancements also enable the identification of subtle patterns and biomarkers associated with early CVD onset, enhancing predictive accuracy beyond traditional statistical methods. Furthermore, these models can support real‐time decision‐making, offering personalized risk assessments and treatment recommendations that improve patient outcomes in cardiovascular healthcare.

### 5.1 Data acquisitions

Traditional health systems often fall short in various dimensions such as wearability, wireless capabilities, durability, and data stability for extended clinical‐quality health data gathering, impacting precise diagnostics.^146^, ^147^, ^148^ This underscores a crucial challenge in leveraging the IoT for personalized healthcare: the imperative for POC devices that are not only affordable and comfortable but also capable of delivering consistent, real‐time health insights.^140^, ^149^, ^150^ Furthermore, advanced sensors integrated with nanomaterials offer promising avenues for accurate data acquisition.^56^, ^57^, ^58^, ^59^, ^77^, ^132^, ^133^, ^134^, ^144^, ^151^, ^152^, ^153^ These key metrics serve as evaluative tools for cardiovascular health, laying the groundwork for a customized healthcare strategy characterized by improved outcomes, user accessibility, superior quality, and economic efficiency.^154^, ^155^, ^156^ These elements are critical in reducing both the prevalence and fatality rates associated with CVDs. Integrating machine learning with advanced data acquisition technologies can transform raw health data into actionable insights, enabling early detection and precise diagnosis of CVDs. By leveraging real‐time data from wearable and IoT‐enabled devices, machine learning models can identify complex patterns and trends in cardiovascular health, providing a proactive approach to disease management and significantly improving patient outcomes.

### 5.2 Translational medicine

Clinical judgment remains indispensable in interpreting machine learning‐generated outcomes. Caution should be exercised in generalizing findings, considering potential biases present in electronic health records (EHRs), which could disproportionately represent specific demographics or health conditions (Figure 5b).^15^ Nevertheless, the transformative potential of machine learning in healthcare is undeniable. When synergized with genomic medicine, phenomapping, and cutting‐edge diagnostic instruments, machine learning has the potential to reshape early detection and treatment across a wide array of cardiovascular ailments.

### 5.3 Accuracy

Despite its data‐centric nature, machine learning is not immune to perpetuating existing biases.^157^, ^158^, ^159^ In critical fields like healthcare, the need for accuracy is paramount. The emerging soft electronics could enhance the accuracy tremendously.^146^, ^160^, ^161^, ^162^ A notable concern is selection bias, which may go unnoticed in machine learning datasets but is controlled for in clinical trials. For instance, a model trained on the EHRs could inaccurately estimate disease prevalence due to biases in patient screening histories. Even more disconcerting, there have been instances where ML algorithms have perpetuated racial and socioeconomic biases, although none have been reported in healthcare to date. This highlights the necessity for stringent measures to eliminate irrelevant biases to ensure reliable clinical decision‐making. Advancements in machine learning for CVDs can improve accuracy by incorporating diverse, unbiased datasets from global populations, ensuring models are representative and applicable across demographics. Additionally, explainable AI techniques can enhance trust and transparency in machine learning‐driven decisions, allowing clinicians to better understand the rationale behind predictions and mitigate potential biases in cardiovascular diagnostics and treatment planning.

### 5.4 Multimodal machine learning

AI has revolutionized various fields like language translation and image recognition, yet its progress in medicine has been slower due to the complexity and high dimensionality of medical data. Recent advances in POC sensors, data aggregation, and the reduced cost of genome sequencing have laid the groundwork for AI‐driven innovations in biomedical discovery, diagnosis, and treatment. Current AI applications in medicine often focus on single data modalities, unlike clinicians who integrate multiple sources for comprehensive decision‐making. However, multimodal AI models that incorporate data across various sources including biosensors, genomics, imaging, and social determinants, hold the potential to transform individualized medicine and real‐time health monitoring (Figure 5c).^140^ Multimodal machine learning can significantly enhance CVDs management by integrating diverse data sources, such as imaging, genomics, and wearable sensor data, to provide a holistic view of patient health. These models can identify complex, cross‐modality patterns associated with CVDs progression, enabling more accurate predictions, personalized treatment strategies, and real‐time monitoring for improved patient outcomes.

### 5.5 Data privacy

The integration of AI and machine learning into healthcare, particularly cardiovascular medicine, face multifaceted obstacles. Among these challenges are issues like improper dichotomization and miscalibration (Figure 5d).^163^, ^164^, ^165^, ^166^ A notable instance involved patient data from the Royal Free London NHS Foundation Trust being provided to Google DeepMind without informed consent, aiming to develop an algorithm for acute kidney injury detection. Instances like this heighten concerns over data transparency, the risk of breaches, and the objectives of for‐profit AI entities in healthcare. The General Data Protection Regulation (GDPR) enacted in 2016 by the European Union has fortified data privacy standards, emphasizing individual consent, breach protocols, and punitive measures for non‐compliance. The escalating dependency on AI for maintaining electronic patient records, which often include both medical and sociodemographic data, highlights the urgency for stringent data protection protocols in the United States where uniform data protection laws are lacking.

### 5.6 Interpretation

The intricacies of modern machine learning, despite its precision, present challenges due to the “black box” phenomenon. Such complexities can be especially concerning in disciplines where decisions can be life‐altering. For example, an automated machine learning‐based cancer diagnostic tool may be as effective as a human physician in detecting malignancies. Yet, its decision‐making process, rooted in DL and intricate abstractions, remains enigmatic. This obscurity raises questions about trust and the potential for algorithmic errors, necessitating periodic human oversight. Explainable AI can help unravel the decision‐making process, providing actionable insights into key predictors of CVDs while ensuring transparency and mitigating potential risks in clinical applications.

## AUTHOR CONTRIBUTIONS

**Kaidong Wang:** Conceptualization; supervision; project administration; writing – original draft; writing – review and editing. **Bing Tan:** Conceptualization; visualization; writing – original draft; writing – review and editing. **Xinfei Wang:** Visualization; writing – review and editing. **Shicheng Qiu:** Writing – review and editing; visualization. **Qiuping Zhang:** Writing – review and editing. **Shaolei Wang:** Visualization; writing – review and editing. **Ying‐Tzu Yen:** Writing – original draft; writing – review and editing. **Nan Jing:** Writing – original draft; writing – review and editing. **Changming Liu:** Visualization; writing – review and editing. **Xuxu Chen:** Conceptualization; project administration; supervision; writing – review and editing. **Shichang Liu:** Conceptualization; project administration; supervision; writing – review and editing. **Yan Yu:** Supervision; writing – review and editing.

## CONFLICT OF INTEREST STATEMENT

The authors declare no conflicts of interest.

## Data Availability

Data sharing not applicable to this article as no datasets were generated or analysed during the current study.
